# Blockade of dual-specificity phosphatase 28 decreases chemo-resistance and migration in human pancreatic cancer cells

**DOI:** 10.1038/srep12296

**Published:** 2015-07-27

**Authors:** Jungwhoi Lee, Jeong Hun Yun, Jungsul Lee, Chulhee Choi, Jae Hoon Kim

**Affiliations:** 1 Faculty of Biotechnology, college of Applied Life Science, SARI, Jeju National University, Jeju-do 690-756, Korea; 2 Department of Bio and Brain Engineering, KAIST, Daejeon 305-701, Korea

## Abstract

Pancreatic cancer remains one of the most deadly cancers, with a grave prognosis. Despite numerous endeavors to improve treatment of the neoplasm, limited progress has been made. In the present study, we investigated the role of dual specificity phosphatase 28 (DUSP28) in relation to anti-cancer drug sensitivity and migratory activity in human pancreatic cancer cells for the first time. Analysis using Universal exPress Codes (UPCs) with the GEO database showed significantly higher DUSP28 mRNA expression in pancreatic cancers. We found that DUSP28 was highly expressed in several human pancreatic cancer cell lines that showed resistance to anti-cancer drugs. Overexpression of DUSP28 decreased anti-cancer drug-sensitivity and enhanced cellular migration via the ERK1/2 pathway in DUSP28-negative cell lines. Knockdown of DUSP28 re-sensitized cells to anti-cancer drugs even at sublethal doses by inducing an apoptotic pathway and significantly reduced migration in DUSP28-positive human pancreatic cancer cell lines. Furthermore, DUSP28-positive cell line (Panc-1) xenograft models were more resistant to gemcitabine treatment than DUSP28-negative cell line (SNU-213) xenograft models. Collectively, these results indicate that DUSP28 plays a key role in drug resistance and migratory activity in human pancreatic cells, and suggest that targeting DUSP28 might have clinical relevance in eradicating malignant pancreatic cancers.

Pancreatic cancer is one of the most deadly and difficult neoplasms to diagnose^1^,^2^. Despite significant improvements in overall survival rates of other cancers during the past few decades, the prognosis of pancreatic cancers has unfortunately remained unchanged^3^,^4^. The main reason for the extremely poor prognosis may be that only a few patients undergo surgical operations on being diagnosed^5^. Another serious feature of pancreatic cancers is the high resistance to conventional cancer therapies, such as chemotherapy and radiation therapy. Moreover, metastatic activity makes pancreatic cancer therapies more difficult. There are no effective treatments to overcome these problems, and no alternative therapies exist for the treatment of refractory or recurrent pancreatic cancers^6^,^7^. Thus, the development of effective therapies for pancreatic cancers is urgently required.

The phosphorylation of proteins is regulated by the reciprocal and specific actions of protein kinases and protein phosphatases. Thus, the roles of phosphatases are important in cell signaling networks as are those of kinases. The members of the protein tyrosine phosphatase (PTP) family are encoded by approximately 100 genes in humans. They can be divided into two main groups; ‘classical’ PTPs and dual-specificity phosphatases (DUSPs). The classical PTPs particularly dephosphorylate proteins with tyrosine residues. The DUSPs can dephosphorylate proteins with serine/threonine residues and tyrosine residue. Both classical PTPs and DUSPs catalyze dephosphorylation by a common mechanism, based on an active site cysteine residue^8^,^9^,^10^.

To date, 25 DUSP genes are listed in the Human Genome Organization database and DUSP17, 20, and 23 overlap with DUSP19, 18, and 25, respectively. DUSPs can be subdivided into three groups by subcellular localization. DUSP1, 2, 4, and 5 are localized to the nucleus (class I), while DUSP6, 7, and 16 are found in the cytoplasm (class II). DUSP8, 9, and 10 can be localized in the nucleus or the cytoplasm (class III). The prevailing substrates of class I and II DUSPs are ERK, p38, and JNK, while class III DUSPs recognize only p38 and JNK as substrates. The strict divisions of substrate specificity and localization make DUSPs suitable targets to understand the complex MAPK signaling networks according to various studies^11^,^12^,^13^. DUSPs also can be classified into two particular groups: typical and atypical DUSPs with or without an additional MAP kinase binding (MKB) domain^14^. Similar to typical DUSPs, atypical DUSPs show specific phosphatase activity via various cellular phenotypes. Previously, DUSP13 has been reported as a positive regulator of apoptosis signal-regulating kinase 1 (ASK1)^15^. DUSP3 and DUSP23 were positively associated in human cervix carcinoma progression and human breast cancer growth, respectively^16^,^17^,^18^. DUSP 1 is associated with resistance of cancer cells to anti-tumor therapies and many DUSPs are receiving attention as potential targets for anti-cancer therapy^19^,^20^. DUSP28 is an atypical DUSP, composed of a single catalytic phosphatase domain specific for phospho-tyrosine residues. DUSPs have a common signature motif, HCXXGXXR, but DUSP28 has a tyrosine in place of the conserved histidine^21^. Recently, atypical roles of DUSP28 have been reported as a regulator of human hepatocellular carcinoma progression^22^. However, there is no previous report about the cellular functions of DUSP28 in malignant pancreatic cancers features.

In the present study, we report the functional role of DUSP28 in pancreatic cancers for the first time. Our results demonstrate that DUSP28 plays a key role in the drug-resistance and migratory activity in human pancreatic cancer cells through the ERK pathway, suggesting that targeting DUSP28 might be a challenging strategy for patients with pancreatic cancer.

## Results

### Expression levels of DUSP28 in human pancreatic cancer tissue and cell lines

**Figure 1:**
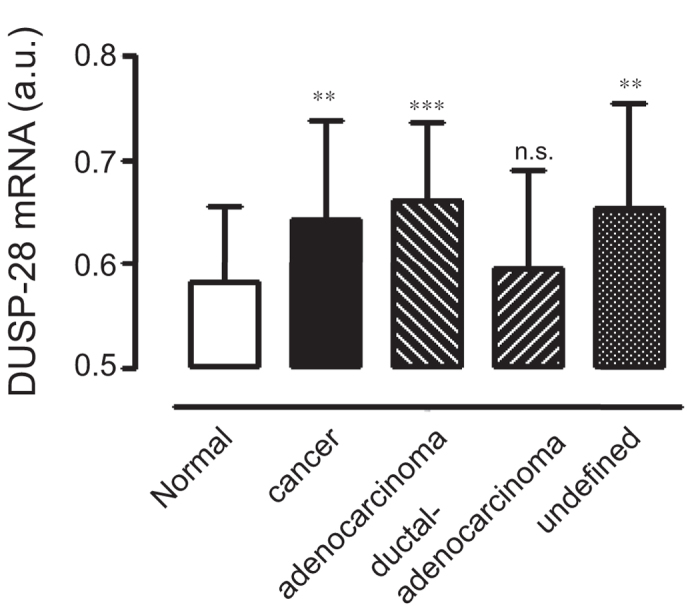
DUSP28 expression using GEO databases. (**a**) Transcriptional levels of DUSP28 in pancreatic cancers and normal pancreatic samples were analyzed by the Gene Expression Omnibus (GEO) databases. Cancer indicates the sum of adenocarcinoma, ductal-adenocarcinoma, and undefined samples (a.u. indicates arbitrary unit using the UPCs method, n.s. means non-significant).

The DUSP28 expression profile was obtained using the public microarray database GEO of human pancreatic cancer samples. As shown in Fig. 1, pancreatic cancers expressed significantly more DUSP28 mRNA than the normal pancreatic samples. Specifically, adenocarcinoma of the pancreas (*n* = 55, GSM# 967641–1053825) and undefined cancers (*n* = 76, GSM# 242823–414974) expressed higher levels of DUSP28 than normal pancreatic samples (*n* = 61, GSM# 388101–463724) and the level of DUSP28 was also slightly higher in the ductal-adenocarcinoma samples (*n* = 91, GSM# 388153–811004), although the difference was not statistically significant.

**Figure 2:**
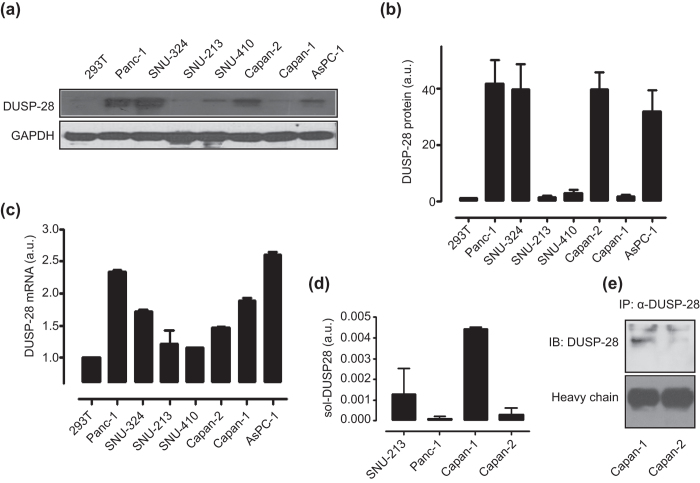
DUSP28 expression in various human pancreatic cancer cells. (**a**) DUSP28 protein in various human pancreatic cancer cells were detected by immunoblot analysis. GAPDH was measured as a control. (**b**) ROIs (regions of interest) of DUSP28 proteins were calculated with GAPDH as control in various human pancreatic cancer cells and 293T cells using ImageJ software (a.u. indicates arbitrary units using values of DUSP28/GAPDH). (**c**) DUSP28 mRNA expression levels of various human pancreatic cancer cells were measured by quantitative real-time PCR (a.u. indicates arbitrary units using values of DUSP28/GAPDH). (**d**) Soluble DUSP28 in SNU-213, Panc-1, Capan-1, and Capan-2 cell culture supernatants was detected by ELISA using an anti-DUSP polyclonal antibody and an anti-DUSP28 monoclonal antibody (a.u. indicates arbitrary units using values of soluble DUSP28/numbers of cells). (**e**) Soluble DUSP28 was precipitated using the anti-DUSP28 antibody in Capan-1 and Capan-2 cell culture supernatants, and then bound DUSP28 was subjected to immunoblot analysis.

We investigated the DUSP28 expression levels in various human pancreatic cancer cell lines by Western blotting and quantitative real-time PCR. The expression patterns of DUSP28 were quite varied. DUSP28 was expressed in Panc-1, SNU-324, Capan-2, and AsPC-1 (Fig. 2a,b) and very weakly detectable in SNU-410, SNU-213, Capan-1, and human embryonic kidney 293T cells under the same conditions (Fig. 2a,b). Quantitative real-time PCR analysis of DUSP28 mRNA expression in the same eight cell lines showed a correlation between mRNA and protein expression, except for Capan-1 cells (Fig. 2c). To examine the discordance between mRNA and protein expression levels in Capan-1 cells, we checked genetic mutations of DUSP28 gene and found no mutation of DUSP28 in Capan-1 cells (data not shown). A high level of soluble DUSP28 was detected in Capan-1 cells culture medium compared with those of SNU-213, Panc-1, and Capan-2 cells using a sandwich ELISA (Fig. 2d). Additionally, the secreted DUSP28 of Capan-1 cells could be detected by immunoprecipitation, compared with that of Capan-2 (Fig. 2e).

These results indicated that DUSP28 expression levels are elevated in many human pancreatic cancers with different levels and forms.

### Differential sensitivities to anti-cancer drugs according to the expression patterns of DUSP28 in human pancreatic cancer cells

The correlation between DUSP28 expression and various anti-cancer drugs was first examined using the CCLE database to investigate the role of DUSP28 in human pancreatic cancers. As shown Table 1, there was negative correlation between DUSP28 expression and anti-cancer drug sensitivity in about 80% (19/24) of human pancreatic cancer cells. However, there was little correlation between anti-cancer drugs sensitivity and DUSP28 expression in other cancer cells, such as hematopoietic tissue, large intestine, lung, prostate, esophagus, breast, bone, stomach, soft tissue, urinary tract, endometrium, skin, liver, central nervous system, thyroid, ovary, kidney, pleura, autonomic ganglia, and upper aerodigestive tract. Furthermore, expression of the other 24 DUSPs showed no meaningful correlation with anti-cancer drugs in pancreatic cancers (data not shown). These results suggested that DUSP28 played a key role in the acquisition of drug resistance in patients with pancreatic cancers.

**Figure 3:**
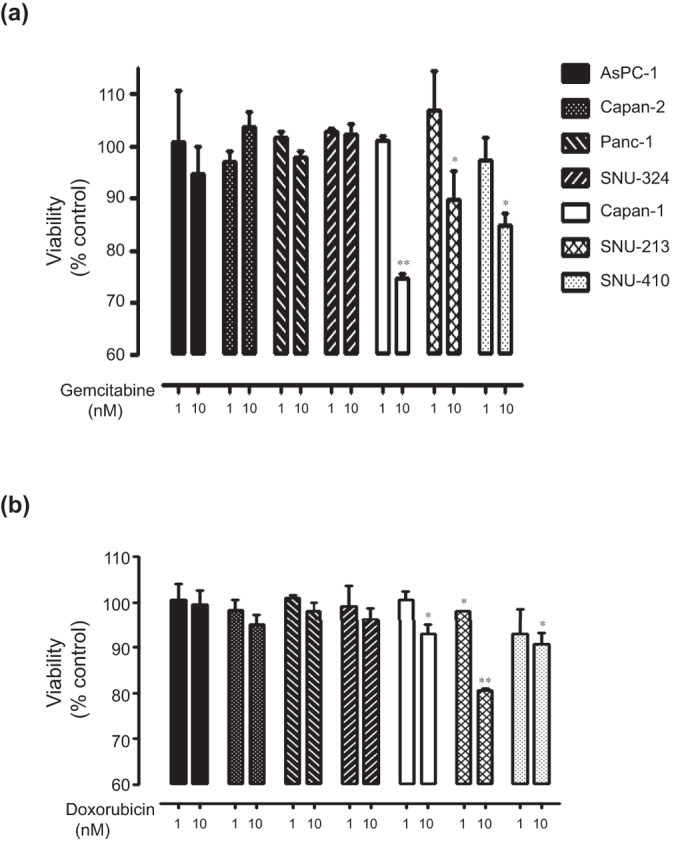
Sensitivities to anti-cancer drugs according to expression patterns of DUSP28. (**a**) Seven human pancreatic cancer cells were incubated with different doses of gemcitabine and doxorubicin for 72 h. (**b**) Viability was measured by the WST-1 assay (*n* = 3; Tukey’s *post hoc* test was applied to significant group effects in ANOVA, p < 0.0001; asterisks indicate a significant difference compared with 0% inhibition, *P < 0.05, **P < 0.01. n.s., non-significant).

Drugs currently used in clinical trials – gemcitabine and doxorubicin – were tested in a dose-dependent manner (0, 1, 10, 100, 1000 nM) to treat seven different human pancreatic cancer cells to assess the role of DUSP28 in drug resistance. Treatment with gemcitabine or doxorubicin showed dose-dependent cytotoxicity in all human pancreatic cancer cells, to differing degrees (data not shown). Treatment with 1 nM gemcitabine did not affect the viability of the cells tested. However, 10 nM of gemcitabine significantly reduced cell viability in Capan-1, SNU-213, and SNU-410 cells, compared with the control, while AsPC-1, Capan-2, Panc-1, and SNU-324 cells were unaffected (Fig. 3a). Similar to gemcitabine, doxorubicin also reduced the number of viable cells at a concentration of 10 nM (Fig. 3b). Different sensitivities to anti-cancer drugs were detected at relatively low concentrations in human pancreatic cancer cells, depending on the DUSP28 expression. Furthermore, there was a tendency for resistance in AsPC-1, Capan-1, Panc-1, and SNU-324 cells even at a relatively high dose (100 nM) of gemcitabine or doxorubicin (data not shown).

These results indicated that chemo-resistance depended on the expression levels of DUSP28 in human pancreatic cancer cells.

### *In vitro* effects of DUSP28 overexpression in human pancreatic cancer cells

**Figure 4:**
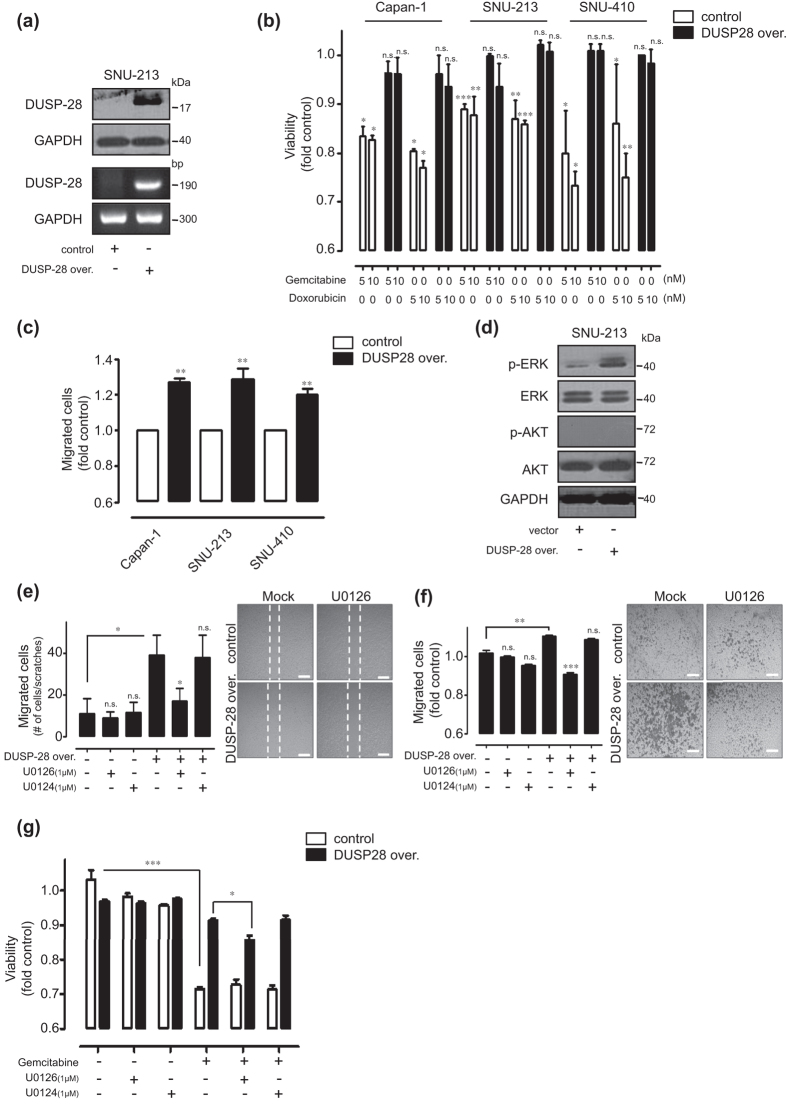
Overexpression of DUSP28 in human pancreatic cancer cells. (**a**) SNU-213 cells were transfected with a vector control or the pcDNA-DUSP28 construct. DUSP28 levels were analyzed by Western blot (top) and RT-PCR (bottom). GAPDH was measured as a control. (**b**) After 48 h of transfections, Capan-1, SNU-213, and SNU-410 cells were s were incubated with different doses of gemcitabine and doxorubicin for an additional 72 h. Viability was measured by the WST-1 assay (*n* = 3; Tukey’s *post hoc* test was applied to significant group effects in ANOVA, p < 0.0001; asterisks indicate a significant difference compared with 0% inhibition, *P < 0.05, **P < 0.01, ***P < 0.001. n.s. means non-significant). (**c**) Capan-1, SNU-213, and SNU-410 cells were transfected with vector control or pcDNA-DUSP28 construct. Migration activities were evaluated using the Transwell migration assay (*P*-value valuated with Student’s *t* test, **P < 0.01). (**d**) After 48 h of transfection with the vector control or pcDNA-DUSP28 construct, SNU-213 cell lysates were subjected to Western blot analysis using antibodies specific for phospho-ERK (Thr202/Tyr204), total ERK, phospho-AKT (S473), and total AKT. GAPDH was measured as a control. (**e**) After 48 h transfection, SNU-213 cells were treated with 1 μM U0126 or U0124. Migration activities were evaluated using the wound-healing assay for 12 h. The quantity of migrated cells is indicated in the graph (left) and representative images (right). Scale bar indicates 100 μm. (**f**) Transwell migration assay for 4 h were performed using the same cells as in Figure 4e (the *P* value was evaluated with Student’s *t* test, *P < 0.05, **P < 0.01, ***P < 0.001. n.s. means non-significant). The quantity of migrated cells indicated as a graph (left) and representative images (right). Scale bar indicates 100 μm. (**g**) After 48 h transfection, SNU-213 cells were incubated with 1 μM of U0126 and U0124 in the absence or presence of 10 nM of gemcitabine for an additional 72 h. Viability was measured by the WST-1 assay (*n* = 3; Tukey’s *post hoc* test was applied to significant group effects in ANOVA, p < 0.0001; asterisks indicate a significa*n*t difference compared with 0% inhibition, *P < 0.05, ***P < 0.001).

To investigate the functional role of DUSP28 in human pancreatic cancer cells, a flag-tagged DUSP28 overexpression system was used in Capan-1, SNU-213, and SNU-410 cells. First, we checked the efficiency of DUSP28 overexpression in these cells. Transient transfection of flag-tagged DUSP28 constructs strongly induced the expression of DUSP28 protein (top) and mRNA (bottom) in SNU-213 (Fig. 4a). Next, we examined the acquisition of chemoresistance in DUSP28-overexpressing Capan-1, SNU-213, and SNU-410 cells at doses of 5 and 10 nM. Surprisingly, overexpressed DUSP28 provided drug resistance to otherwise lethal doses of gemcitabine and doxorubicin in these cells (Fig. 4b).

The migration activities were also assessed in human pancreatic cancer cells with enhanced DUSP28 expression. Overexpression of DUSP28 markedly induced migration activity in Capan-1, SNU-213, and SNU-410 cells (Fig. 4c). In DUSP28 overexpressing Capan-1 cells, we could detect the increased levels of DUSP28 protein in both cell lysates and culture supernatants (Supplementary Figure 1).

We further examined the signal transduction events that were particularly activated by overexpression of DUSP28. Overexpressed DUSP28 induced ERK1/2 phosphorylation; while the same conditions had no effect on AKT phosphorylation (Fig. 4d). To confirm the involvement of the ERK1/2 pathway in the acquisition of functions by DUSP28 overexpression, DUSP28-overexpressing SNU-213 cells were incubated in the absence or presence of an ERK1/2 inhibitor (U0126; 1 μM) or an inactive structural analog (U0124; 1 μM) in wound-healing assay. Treatment with U0126 significantly inhibited the migration of DUSP28-overexpressing SNU-213 cells. However, there was no effect of U0124 treatment under same conditions (Fig. 4e). Similarly, treatment with U0126 markedly inhibited DUSP28 mediated migration using a Transwell migration assay (Fig. 4f). Additionally, treatment with UO126 reduced the acquired drug-resistance, compared with U0124 treatment in DUSP28-overexpressing SNU-213 cells (Fig. 4g).

### *In vitro* effect of DUSP28 blockade in pancreatic cancer cells

**Figure 5:**
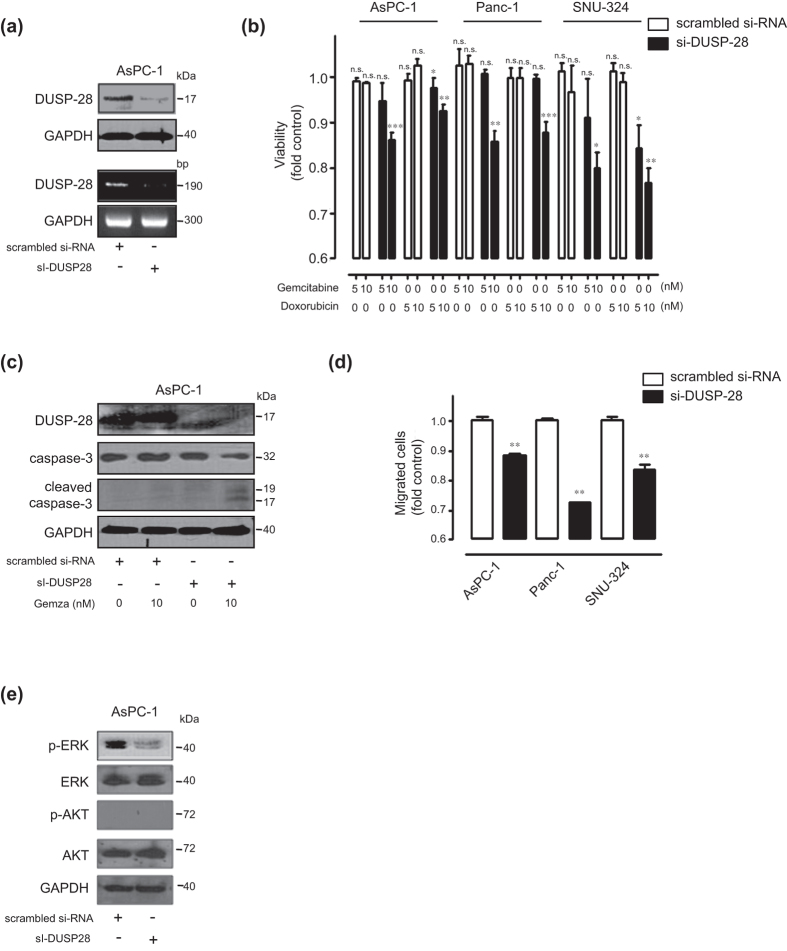
Knock-down of DUSP28 in intrinsic chemo-resistant pancreatic cancer cells. (**a**) AsPC-1 cells were transfected with scrambled or DUSP28-specific siRNA (sc-94445). After 72 h of transfection, DUSP28 levels were analyzed by Western blot (top) and RT-PCR (bottom). GAPDH was measured as a control. (**b**) AsPC-1, Panc-1, and SNU-324 cells were transfected with scrambled or DUSP28-specific siRNA. At 48 h post-transfection, cells were incubated with different doses of gemcitabine and doxorubicin for an additional 72 h. Viability was measured by the WST-1 assay (*n* = 3; Tukey’s *post hoc* test was applied to significant group effects in ANOVA, p < 0.0001; asterisks indicate a significant difference compared to 0% inhibition, **P < 0.01, ***P < 0.001. n.s. means non-significant). (**c**) AsPC-1 cells were transfected with scrambled or DUSP28-specific siRNA. At 48 h post-transfection, cells were incubated with 10 μM of gemcitabine for an additional 72 h. Cell lysates were subjected to Western blot analysis using antibodies specific for DUSP28, total caspase-3, cleaved caspase-3, and GAPDH. (**d**) AsPC-1, Panc-1, and SNU-324 cells were incubated with scrambled or DUSP28-specific siRNA for 48 h. DUSP28-mediated cell migration was measured by the Transwell migration assay for 6 h (*P*-value evaluated with Student’s *t* test). (**e**) AsPC-1 cells were transfected with scrambled or DUSP28-specific siRNA. After 48 h of transfection, AsPC-1 cell lysates were subjected to immunoblot analysis using antibodies specific for phospho-ERK (Thr202/Tyr204), total ERK, phospho-AKT (S473), and total AKT. GAPDH was measured as a control.

We next tested whether reduction of DUSP28 expression by siRNA transfection could re-acquire sensitivity to the anti-cancer drugs gemcitabine and doxorubicin. Compared with the scrambled siRNA-transfected cells, cells transfected with two different DUSP28-specific siRNAs (DUSP28 siRNA) showed significantly decreased DUSP28 protein (top) and mRNA (bottom) levels (Fig. 5a and Supplementary Fig. 3a) and reduced cell viability with non-detrimental doses of gemcitabine and doxorubicin in AsPC-1, Panc-1, and SNU-324 cells (Fig. 5b and Supplementary Fig. 3b). Capan-2 cells showed strong resistance to treatment with gemcitabine and doxorubicin compared with AsPC-1, Panc-1, and SNU-324 cells. However, knock-down of DUSP28 re-sensitized cells to gemcitabine and doxorubicin, even in Capan-2 cells (Supplementary Figure 2). Co-treatment with DUSP28 siRNA transfection and anti-cancer drugs in pancreatic cancer cells resulted in the typical morphology of apoptotic cell death. Thus, we further investigated the involvement of caspase-3. As expected, co-treatment of gemcitabine with knock-down of DUSP28 induced cleavage of caspase-3, while treatment of gemcitabine with scrambled siRNA transfection resulted in no cleavage of procaspase-3 in AsPC-1 cells (Fig. 5c). Similar results were obtained using Panc-1 and SNU-324 cells (data not shown). We assessed migration activities of the cells following DUSP28 siRNA transfection in AsPC-1, Panc-1, and SNU-324 cells. Blockade of DUSP28 expression markedly diminished migratory activity of AsPC-1, Panc-1, and SNU-324 cells (Fig. 5d and Supplementary Fig. 3c). To confirm the mechanism involved in drug resistance and migration activity, the signaling pathway involved was confirmed by immunoblot analysis. DUSP28 siRNA transfection reduced basal levels of ERK1/2 phosphorylation in DUSP28 siRNA-transfected AsPC-1 cells (Fig. 5e and Supplementary Fig. 3d).

### *In vivo* effects of gemcitabine treatment on DUSP28 expression levels

**Figure 6:**
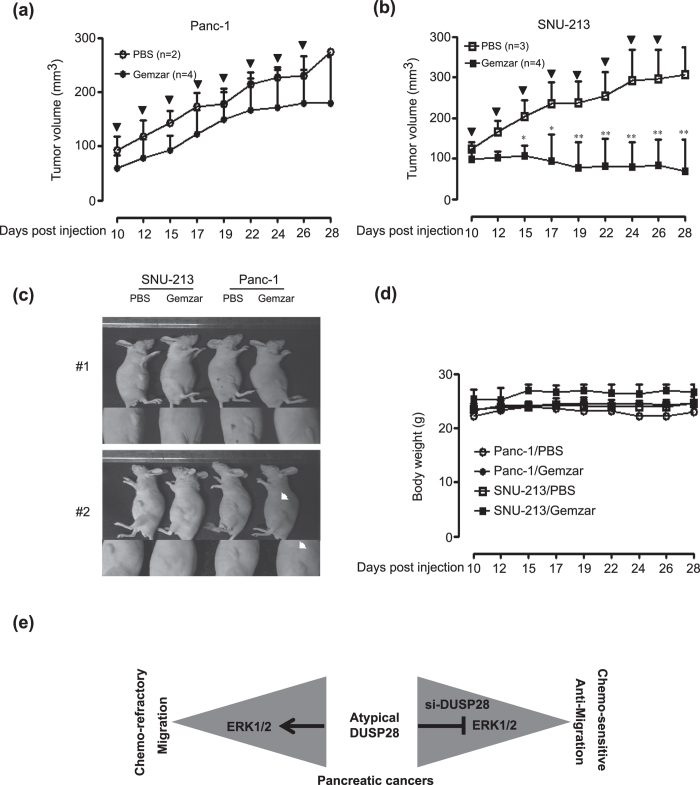
*In vivo* effects of gemcitabine treatment on DUSP28 expression. (**a**) Effects of gemcitabine injection in Panc-1 xenograft models (control group: *n* = 2, gemcitabine group: *n* = 4) were measured for 28 days using the formula: V = 0.523 LW^2^ (L = length, W = width). Bold arrows indicate the time of gemcitabine injection (Tukey’s *post hoc* test was applied to sig*n*ificant group effects in ANOVA, p < 0.0001). (**b**) Effects of gemcitabine injection in SNU-213 xenograft models (control group: *n* = 3, gemcitabine group: *n* = 4) were measured for 28 days using the formula: V = 0.523 LW^2^ (L = length, W = width). Bold arrows indicate the time of gemcitabine injection (Tukey’s *post hoc* test was applied to significant group effects in ANOVA, p < 0.0001; asterisks indicate a significant difference between the PBS-injected group and the gemcitabine-injected group, *P < 0.05, **P < 0.01). (**c**) Representative image of each group indicated the effects of gemcitabine injection. White arrows indicate the newly generated tumor from the Panc-1 cell-injected tumor. (**d**) The body weight in each group was measured regularly. (**e**) A diagram to illustrate the role of DUSP28 in human pancreatic cancers.

To investigate the functional roles of DUSP28 in drug resistance *in vivo*, we prepared DUSP28-positive and -negative pancreatic cancer models using Panc-1 and SNU-213 cells, respectively. The tumor-laden mice were injected ip with PBS or gemcitabine when the tumors reached an average size of approximately 100 mm^3^. PBS-treated Panc-1 xenograft tumors grew to an average size of 275.01 ± 6.17 mm^3^ by 28 days after transplantation and there was no significant difference in the gemcitabine-treated Panc-1 xenograft models (Fig. 6a,c), while PBS-treated SNU-213 xenograft tumors grew to an average size of 306.6 ± 68.6 mm^3^ by 28 days after transplantation and gemcitabine-treated xenograft models grew to an average size of 69.71 ± 77.13 mm^3^ (Fig. 6b,c). There was no significant weight loss in either the control or gemcitabine treatment groups in the Panc-1 and SNU-213 xenograft models (Fig. 6c,d).

Collectively, these results indicated that DUSP28 is a key molecule responsible for the chemo-resistance and migratory activity, through the ERK1/2 signaling pathway, and targeting DUSP28 with conventional chemotherapy might be an effective strategy for treatment of pancreatic cancers (Fig. 6e).

### Discussion

An advanced understanding of the molecular and cellular basis of pancreatic cancers may facilitate potential clinical strategies. However, new attempts have not been successful in improving clinical outcomes as yet^23^. This non-responsiveness might originate from the intrinsic or acquired resistance behaviors to chemotherapies via various genetic and cellular mechanisms^24^,^25^, epithelial-mesenchymal transition^26^,^27^, hypoxia^28^,^29^, and pancreatic cancer stem cells^30^,^31^, as reported previously.

In this report, we demonstrated that pancreatic cancers show relatively high expression of DUSP28 versus normal pancreatic samples using the public microarray database GEO. Its specific expression was more meaningful because the comparative analyses included various types of tumors, such as brain, breast, colon, liver, lung, ovary, prostate, skin, and tongue. DUSP28 expression was not significant in almost all tumors compared with normal samples (data not shown). We also confirmed that human pancreatic cancer cell lines have relatively varied levels of DUSP28 mRNA and protein, compared with human kidney 293T cells. Also, DUSP28 was detected in Capan-1 cell culture supernatant. Recent reports have proposed that secreted soluble molecules can be detected as potential biomarkers in pancreatic cancers *in vitro* and *in vivo*^32^,^33^. However, our finding suggested that DUSP28 can be detected in GBM cell culture supernatant using readily accessible techniques, such as ELISA and immunoprecipitation, for the first time. The convenient detection of a soluble form of DUSP28 might be a potential biomarker for pancreatic cancers. However, more research is required to reveal the specific conditions and mechanism involved in the release of DUSP28 from pancreatic cancers.

There was a strong negative correlation between DUSP28 expression and various anti-cancer drugs, according to the CCLE data base. In contrast, there was no notable correlation between the expression of other DUSPs and drug sensitivities excluding DUSP16. However, DUSP16 had similar expression levels in both pancreatic cancers and normal pancreatic samples (data not shown). Additionally, we showed that drug sensitivity was demarcated by basal expression patterns of DUSP28 in human pancreatic cancer cell lines. These results suggested that DUSP28 might be a “messenger” molecule to anti-cancer drugs via operating chemo-resistance in human pancreatic cancers. Furthermore, transient overexpression of DUSP28 conferred chemoresistance to DUSP28-negative pancreatic cancer cells, including cell migration activity, via the ERK1/2 signaling pathway. Downregulation of DUSP28 rendered cells sensitive to anti-cancer drugs again, through the caspase-3 apoptotic pathway, and it also decreased the migration activity of pancreatic cancer cells. Together, these results indicate that DUSP28 plays specialized roles in chemo-resistance and migration in human pancreatic cancer cells via the ERK1/2 pathway.

Although high level expression of DUSP28 was reported to induce p38 activation, resulting in an increase in S phase in hepatocellualr carcinoma cells^22^, DUSP28-related chemo-resistance was via the ERK1/2 signaling pathway consistent with previous reports^34^,^35^. However, currently, gaps in understanding the mechanism of ERK1/2 activation and the classical role of DUSPs as the regulators of dephosphorylation in the MAPK signaling network remain.

Several atypical DUSPs have been reported in various cancer phenotypes, such as apoptosis, proliferation, cell cycle, and chemo-resistance^36^,^37^. A previous report showed that DUSP26 was a phosphatase for the p53 tumor suppressor, inhibiting p53 activity^38^. Another report demonstrated that enhanced DUSP12 induced cellular migration and protection from apoptosis through the upregulation of two validated oncogenes: *i.e.*, integrin alpha 1 and the hepatocyte growth factor receptor, c-met^39^. Proliferating cell nuclear antigen (PCNA) was upregulated or decreased in DUSP28 overexpression or knock-down, even though there was no viability difference with DUSP28 expression levels in our study. PCNA might be involved in chemo-resistance in human pancreatic cancer cells as reported previously^40^,^41^. We also showed that drug sensitivity was differently affected by DUSP28 expression levels in human pancreatic cancer *in vivo*. Remarkably, newly generated tumors were observed exclusively in the area remote from the injection site in Panc-1 xenograft models, but not in SNU-213 xenograft models (data not shown). This phenotype suggested that DUSP28 might be a key molecule to regulate chemo-resistance and micro-metastasis in pancreatic cancers. Further study is required to reveal the mechanism involved in the unique role of DUSP28 not limited to phosphatase activity.

In summary, our report provides a background for the disappointing prognosis in pancreatic cancer patients undergoing chemotherapy. We also suggest a potentially therapeutic strategy to overcome the chemo-resistance and local metastasis in pancreatic cancers. Our research provides further insight to control the malignancy involving DUSP28 as atypical positive feedback signaling and offers the possibility of DUSP28 as a new biomarker for pancreatic cancers.

## Materials and Methods

### Gene expression analysis

Microarray expression profiles were obtained from the Gene Expression Omnibus (GEO) public microarray database. We integrated data sets independently obtained from several research groups using the absolute normalization method SCAN.UPC^42^. We restricted the integration to Affymetrix Human Genome U133 Plus 2.0 Array platform (GPL570) because the normalization method is dependent on the total number of probes and GPL570 has more probes than GPL96 and GPL97. All data were normalized by the default option of SCAN.UPC. The histological type of each sample was assigned according to sample annotation in GEO. Samples without histological information were assigned as ‘undefined.’ In total, eight data sets were used: GSE9599, GSE15471, GSE16515, GSE17891, GSE32676, GSE39409, GSE42952, and GSE46385. Correlations between various anti-cancer drugs and DUSP28 expression levels were analyzed with the Cancer Cell Line Encyclopedia (CCLE) public database.

### Cell culture and reagents

Human pancreatic cancer cell lines (AsPC-1, Capan-1, Capan-2, Panc-1, SNU-213, SNU-324, and SNU-410) were purchased from the Korean Cell Line Bank (KCLB, Seoul, Korea). A human kidney cell line (293T) was obtained from the American Type Culture Collection (Manassas, VA). The cells were grown in DMEM (Panc-1 and 293T) or RPMI 1640 (AsPC-1, Capan-1, Capan-2, SNU-213, SNU-324, and SNU-410) medium supplemented with 10% fetal bovine serum (Gibco-BRL, Gaithersburg, MD, USA), 1 × 10^5^ unit/L penicillin, and 100 mg/L streptomycin (Invitrogen, Carlsbad, CA, USA) at 37 °C in a humidified atmosphere containing 5% CO_2_. Polyclonal antibodies for DUSP28 were obtained from Santa Cruz Biotechnology (Santa Cruz, CA, USA) and monoclonal antibodies for DUSP28 were purchased from Calbiochem (La Jolla, CA). Antibodies for caspase-3, cleaved caspase-3, phospho-ERK (Thr202/204), ERK, phospho-AKT (Ser473), AKT, and GAPDH were purchased from Cell Signaling Technology (Beverly, MA, USA). Doxorubicin and gemcitabine were from Sigma-Aldrich (St. Louis, MO, USA). U0126 and U0124 were purchased from Calbiochem (La Jolla, CA).

### Enzyme-linked immunosorbent assay (ELISA)

Soluble DUSP28 protein from cell-free culture supernatant was determined using a common sandwich-ELISA as described previously^43^.

### Immunoprecipitation

Capan-1 and Capan-2 cells were seeded in a 60-mm dish. After 2 days, culture medium was substituted with fresh medium containing 0.2% serum and cells were incubated for an additional 24 h. Supernatant aliquots were incubated with anti-DUSP28 antibodies, followed by the addition of Protein G agarose (Amersham Bioscience, Little Chalfont, UK). Bound proteins were subjected to SDS-PAGE and Western blotting.

### Transfection of siRNA

Transfection of siRNAs was performed using the effectene reagent (Qiagen, Hilden, Germany) according to the manufacturer’s protocol^43^. Oligonucleotides specific for DUSP28 (sc-94445 and 1120164a/b) were purchased from Santa Cruz Biotechnology (Santa Cruz, CA, USA) and Bioneer (Daejeon, Korea) respectively. Scrambled control (sc-37007) was purchased from Santa Cruz Biotechnology. The efficacy of siRNA transfection was confirmed by Western blot analysis of corresponding proteins.

### Measurement of cell viability

To evaluate cell viability with treatment of gemcitabine or doxorubicin, the WST-1 reagent (Nalgene, Rochester, NY) was used as described previously^43^. After a 10-min incubation at room temperature, the absorbance was measured at 450 nm using a microplate reader (Bio-Rad, Richmond, CA, USA).

### Transwell migration assay

Migration assays were performed using 24-well Transwells (Corning, MA, USA), as described previously^43^. Cells were applied to the upper chamber containing RPMI without serum, and the cells that migrated to the underside of the filter in 6 h were stained. The eluted dye was measured at 560 nm in an ELISA reader (Bio-Rad, Richmond, CA).

### Western blot analysis

To evaluate the protein levels of DUSP28 in human pancreatic cancer cells, Western blot analysis was performed as described previously^44^. Glyceraldehyde 3-phosphate dehydrogenase (GAPDH) was used to ensure equal protein loading.

### RNA preparation, reverse transcript, and quantitative real-time polymerase chain reaction

Total RNA was extracted using the TRIzol reagent (Invitrogen, Carlsbad, CA, USA) and converted to cDNA using a RNA PCR kit (Takara Bio Inc, Japan) according to the manufacturer’s protocol. RT-PCR and quantitative real-time PCR analysis were performed using a Takara RT-PCR kit and StepOne Real-Time PCR system (Applied Biosystems) according to the manufacturers’ protocols. Expression levels were normalized to GAPDH gene levels. Primers for human DUSP28 were obtained from Bioneer (Daejeon, Korea) and GAPDH gene primers were designed using Primer Express 3.0 (Applied Biosystems). Primer sequences for RT-PCR were as follows: GAPDH (forward primer, 5′-TCACTGGCATGGCCTTCCGTG-3′; reverse primer, 5′-GCCATGAGGTCCACCACCCTG-3′) and DUSP28 (forward primer, 5′-GCGGGATCCATGGGACCGGCAGAAGCTGGGCG-3′; reverse primer, 5′-CCCGCTCGAGTCAAGCCTCAGGGCCCAACCCTAA-3′). Primer sequences for quantitative real-time PCR were as follows: GAPDH (forward primer, 5′-TCACTGGCATGGCCTTCCGTG-3′; reverse primer, 5′-GCCATGAGGTCCACCACCCTG-3′) and DUSP28 (forward primer, 5′-CCCGTGTTCGACGACCC-3′; reverse primer, 5′-GCGCTCTTCACCATCTG-3′).

### Construct for over-expression of DUSP28

For overexpression of DUSP28 in human pancreatic cancer cells, plasmids expressing flag-tagged DUSP28 were constructed with a sequence-specific forward primer and a reverse primer bearing the flag sequence, and then ligated into *Nhe*I and *Xho*I sites of pcDNA 3.1 vector (Invitrogen, Carlsbad, CA, USA). The primer sequences for DUSP28 were as follows: forward primer, 5′-GCTAGCTAGCCACCATGGGACCGGCAGAAGCTGG-3′; reverse primer, 5′-GCCGCTCGAGTTACTTGTCATCGTCGTCCTTGTAGTC AGC CTC AGG GCC CAA CCC TA-3′.

### Wound-healing assay

Control vector or flag-tagged DUSP28 construct-transfected SNU-213 cells were maintained in medium containing 0.2% serum for 24 h before being scratched in a straight line with a micropipette tip. Then, 12 h later, unilateral migration was estimated by counting the number of cells entering the scratched region, as described previously^44^.

### Xenograft tumor model

BALB/c nude mice were obtained from Orient (Seongnam, Korea) at 6–8 weeks old. SNU-213 (1 × 10^7^) and Panc-1 cells (1 × 10^7^) were injected subcutaneously into both sides of the flank. Once the tumors achieved a size of approximately 100 mm^3^, mice were randomized to two experimental groups to receive PBS or gemcitabine (10 mg/kg). Tumor volume was calculated using the following formula: V = 0.523 LW^2^ (L = length, W = width). Body weight was recorded regularly. Animal care and experiments were carried out in accordance with guidelines approved by the animal bioethics committee of Jeju National University.

### Statistical analysis

All data are presented as means ± standard deviation. Levels of significance for comparisons between two independent samples were determined by Student’s t-test. Groups were compared by one-way ANOVA with Tukey’s *post hoc* test applied to significant main effects (SPSS 12.0 K for Windows; SPSS Inc., Chicago, IL).

## Additional Information

**How to cite this article**: Lee, J. *et al.* Blockade of dual-specificity phosphatase 28 decreases chemo-resistance and migration in human pancreatic cancer cells. *Sci. Rep.* **5**, 12296; doi: 10.1038/srep12296 (2015).

## Supplementary Material

Supplementary Figure 1

Supplementary Figure 2

Supplementary Figure 3

## Figures and Tables

**Table 1:** Drug-sensitivities correlated with DUSP-28 expression levels in the CCLE databases.

**Drug name**	**correlation**	**average**	**variation**
Paclitaxel	−0.569787	0.6412	0.0257709
RAF-265	−0.565217	0.6521	0.0279145
17-AAG	−0.518681	0.6412	0.0257709
Irinotecan	−0.384211	0.6407	0.0257226
Lapatinib	−0.363985	0.6412	0.0257709
Panobinostat	−0.358511	0.6412	0.0257709
TKI258	−0.333881	0.6412	0.0257709
Topotecan	−0.330049	0.6412	0.0257709
L-685458	−0.243372	0.6412	0.0257709
PD-0332991	−0.230443	0.6431	0.0266271
PF2341066	−0.209633	0.6412	0.0257709
Erlotinib	−0.186645	0.6412	0.0257709
AZD0530	−0.148878	0.6412	0.0257709
LBW242	−0.133005	0.6412	0.0257709
TAE684	−0.115490	0.6412	0.0257709
ZD-6474	−0.110501	0.6346	0.0250656
PHA-665752	−0.107886	0.6412	0.0257709
AZD6244	−0.047619	0.6412	0.0257709
PD-0325901	−0.038314	0.6412	0.0257709
Sorafenib	0.025177	0.6412	0.0257709
Nilotinib	0.054545	0.6327	0.0245046
Nutlin-3	0.100178	0.6412	0.0257709
PLX4720	0.126374	0.6346	0.0250656
AEW541	0.221128	0.6412	0.0257709

Correlation of sensitivities to various anti-cancer drugs with DUSP28 expression levels in 43 different pancreatic cancer cells.
