# Supplementary material for: Blockade of dual-specificity phosphatase 28 decreases chemo-resistance and migration in human pancreatic cancer cells
Source: Sci Rep. 2015 Jul 27;5:12296. doi: 10.1038/srep12296 (PMC4515742; doi:10.1038/srep12296)
Supplement: Supplementary Figure 1 [file srep12296-s1.pdf]

Blockade of dual-specificity phosphatase 28 decreases chemo-resistance and migration in human pancreatic cancer cells

Authors and Affiliations

Jungwhoi Lee[1], Jeong Hun Yun[1], Jungsul Lee[2], Chulhee Choi[2], and Jae Hoon Kim[1]*

[1]Department of Applied Life Science, SARI, Jeju National University, Jeju-do 690-756, Korea,

[2]Department of Bio and Brain Engineering, KAIST, Daejeon 305-701, Korea

Running Title

Roles of DUSP28 in human pancreatic cancer

*Address correspondence to Kim Jae Hoon, Department of Applied Life Science, Jeju National University, 102 Jejudaehak-ro, Jeju-si, Jeju-do 690-756, Republic of Korea. Tel: +82-64-729-8556; Fax: +82-64-756-3351; E-mail: kimjh@jejunu.ac.kr

Supplementary Figure 1.

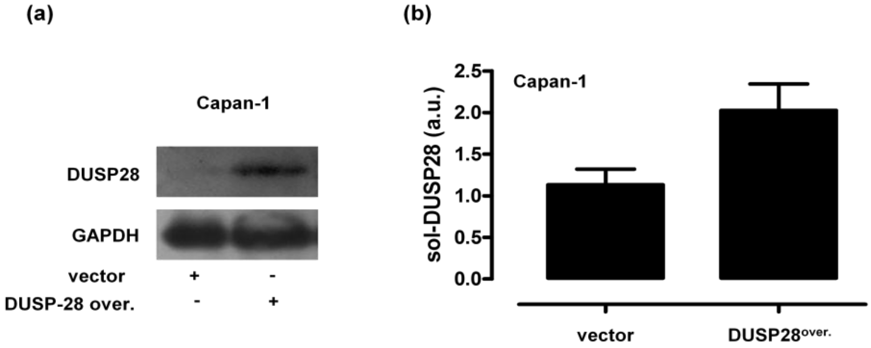

DUSP28 was expressed in-and-out of Capan-1 cells byDUSP28 over-expression. (A) DUSP28 protein was analyzed by immuneblot using anti-DUSP28 antibody in DUSP28 over-expressed Capan-1 cells. (B) Soluble DUSP28 in DUSP28 over-expressed Capan-1 cells cultured supernatants was detected by ELISA using anti-DUSP polyclonal antibody and anti-DUSP28 monoclonal antibody (a.u. indicates arbitrary unit using values of soluble DUSP28/numbers of cells).
