# Supplementary material for: Blockade of dual-specificity phosphatase 28 decreases chemo-resistance and migration in human pancreatic cancer cells
Source: Sci Rep. 2015 Jul 27;5:12296. doi: 10.1038/srep12296 (PMC4515742; doi:10.1038/srep12296)
Supplement: Supplementary Figure 2 [file srep12296-s2.pdf]

Blockade of dual-specificity phosphatase 28 decreases chemo-resistance and migration in human pancreatic cancer cells

Authors and Affiliations

Jungwhoi Lee[1], Jeong Hun Yun[1], Jungsul Lee[2], Chulhee Choi[2], and Jae Hoon Kim[1]*

[1]Department of Applied Life Science, SARI, Jeju National University, Jeju-do 690-756, Korea,

[2]Department of Bio and Brain Engineering, KAIST, Daejeon 305-701, Korea

Running Title

Roles of DUSP28 in human pancreatic cancer

*Address correspondence to Kim Jae Hoon, Department of Applied Life Science, Jeju National University, 102 Jejudaehak-ro, Jeju-si, Jeju-do 690-756, Republic of Korea. Tel: +82-64-729-8556; Fax: +82-64-756-3351; E-mail: kimjh@jejunu.ac.kr

Supplementary Figure 2.

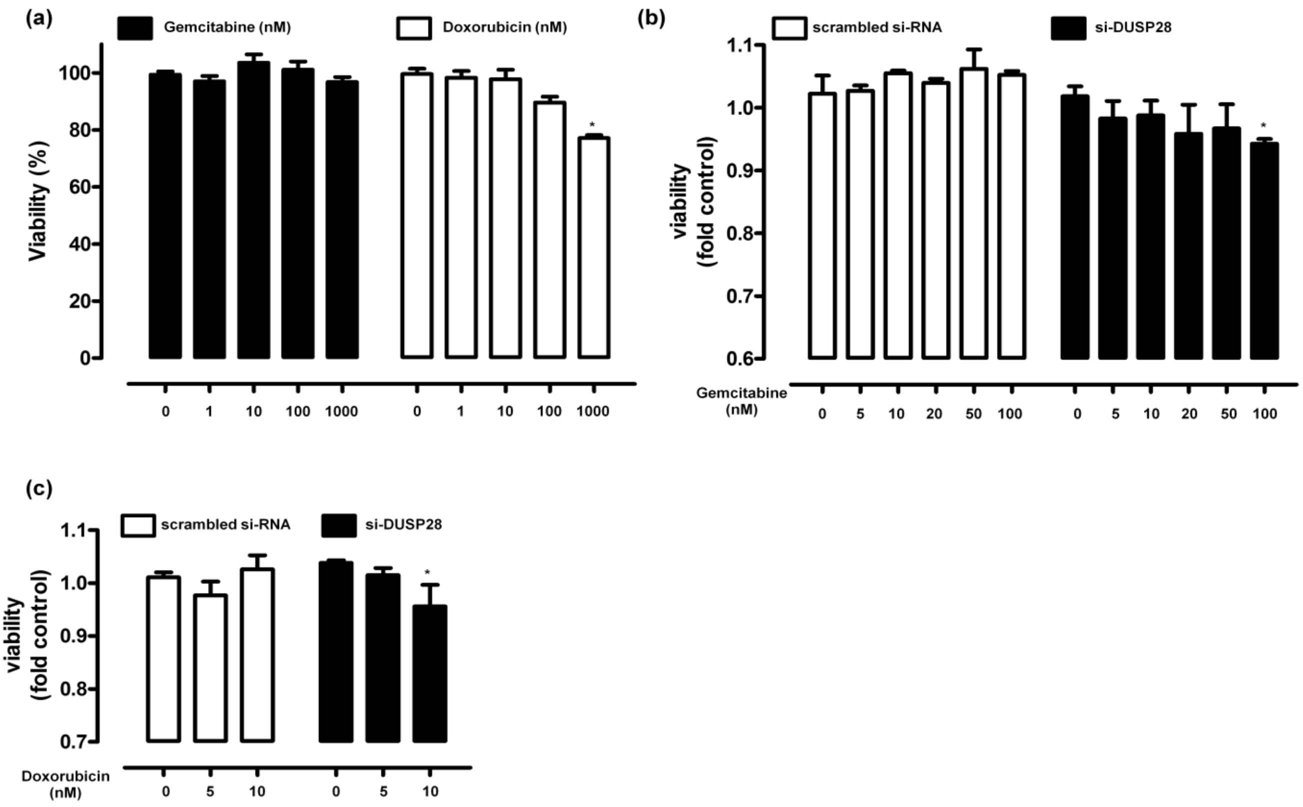

The sensitivity to anti-cancer drugs in Capan-2 cells. (A) Capan-2 cells were incubated with different doses of gemcitabine and doxorubicin for 72 h. Viability was measured by the WST-1 assay (n=3; Tukey's *post hoc* test was applied to significant group effects in ANOVA, $p<0.0001$; asterisks indicate a significant difference compared to 0% inhibition, *$P<0.05$). (B) Capan-2 cells were transfected with scrambled or DUSP28 specific siRNA. 48 h post-transfection, cells were incubated with different doses of gemcitabine and doxorubicin (C) for an additional 72 h. Viability was measured by the WST-1 assay (n=3; Tukey's *post hoc* test was applied to significant group effects in ANOVA, $p<0.0001$; asterisks indicate a significant difference compared to 0% inhibition, *$P<0.05$).
