# Supplementary material for: Blockade of dual-specificity phosphatase 28 decreases chemo-resistance and migration in human pancreatic cancer cells
Source: Sci Rep. 2015 Jul 27;5:12296. doi: 10.1038/srep12296 (PMC4515742; doi:10.1038/srep12296)
Supplement: Supplementary Figure 3 [file srep12296-s3.pdf]

Blockade of dual-specificity phosphatase 28 decreases chemo-resistance and migration in human pancreatic cancer cells

Authors and Affiliations

Jungwhoi Lee[1], Jeong Hun Yun[1], Jungsul Lee[2], Chulhee Choi[2], and Jae Hoon Kim[1]*

[1]Department of Applied Life Science, SARI, Jeju National University, Jeju-do 690-756, Korea,

[2]Department of Bio and Brain Engineering, KAIST, Daejeon 305-701, Korea

Running Title

Roles of DUSP28 in human pancreatic cancer

*Address correspondence to Kim Jae Hoon, Department of Applied Life Science, Jeju National University, 102 Jejudaehak-ro, Jeju-si, Jeju-do 690-756, Republic of Korea. Tel: +82-64-729-8556; Fax: +82-64-756-3351; E-mail: kimjh@jejunu.ac.kr

Supplementary Figure 3.

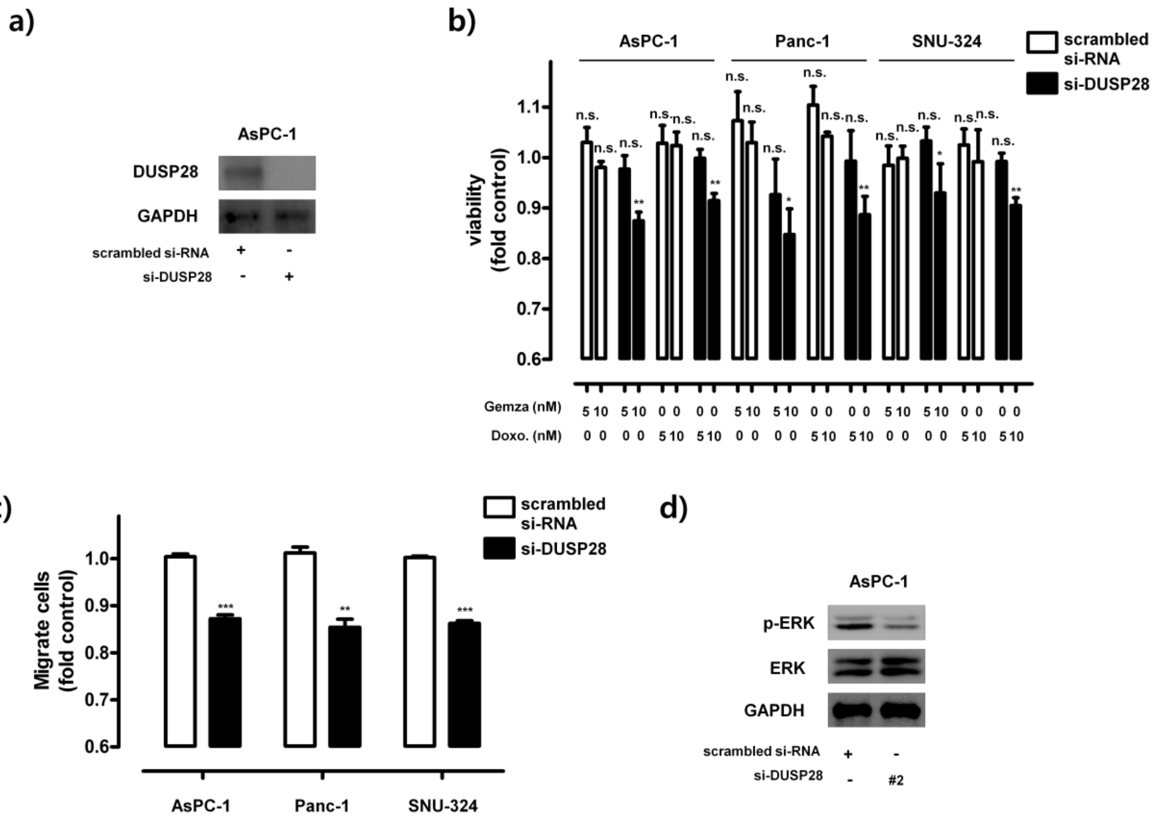

Knock-down of DUSP28 in intrinsic chemo-resistant pancreatic cancer cells

(a) AsPC-1 cells were transfected with scrambled or DUSP28-specific siRNA (1120164a/b) purchased from Bioneer (Daejon, Korea). After 72 h of transfection, DUSP28 levels were analyzed by Western blot. GAPDH was measured as a control. (b) AsPC-1, Panc-1, and SNU-324 cells were transfected with scrambled or DUSP28-specific siRNA. At 48 h post-transfection, cells were incubated with different doses of gemcitabine and doxorubicin for an additional 72 h. Viability was measured by the WST-1 assay (n = 3; Tukey's *post hoc* test was applied to significant group effects in ANOVA, $p < 0.0001$; asterisks indicate a significant difference compared to 0% inhibition, *$P < 0.05$, **$P < 0.01$, ***$P < 0.001$. n.s. means non-significant). (c) AsPC-1, Panc-1, and SNU-324 cells were incubated with scrambled or DUSP28-specific siRNA for 48 h. DUSP28-mediated cell migration was measured by the

Transwell migration assay for 6 h (*P*-value evaluated with Student's *t* test). (d) AsPC-1 cells were transfected with scrambled or DUSP28-specific siRNA. After 48 h of transfection, AsPC-1 cell lysates were subjected to immunoblot analysis using antibodies specific for phospho-ERK (Thr202/Tyr204), total ERK, and GAPDH.
